# Cell Invasion in Collagen Scaffold Architectures Characterized by Percolation Theory

**DOI:** 10.1002/adhm.201500197

**Published:** 2015-04-16

**Authors:** Jennifer C Ashworth, Marco Mehr, Paul G Buxton, Serena M Best, Ruth E Cameron

**Affiliations:** Department of Materials Science and Metallurgy, University of Cambridge27 Charles Babbage Road, Cambridge, CB3 0FS, UK E-mail: rec11@cam.ac.uk; Geistlich Pharma AG, Core TechnologiesBahnhofstrasse 40, CH-6110, Wolhusen, Switzerland

**Keywords:** cell invasion, collagen scaffolds, interconnectivity, percolation, scaffold characterization

Collagen scaffolds are biological templates for the regeneration of damaged or diseased tissues. Since cell invasion into these porous scaffolds is vital for healthy tissue regeneration, the characteristics that influence cellular response must be accounted for in scaffold design. For instance, characterization of mean pore size has revealed that cell migration is highly influenced by scaffold structure.[1,2] However, in order to invade at all, cells require a pathway of connected pores: a characteristic that is not described by mean pore size. The extent to which such pathways are present is termed the interconnectivity, and this term may be used to include the number, size, and shape of these pathways. Interconnectivity also has acknowledged importance for nutrient supply and the formation of vascularized tissue deep within the scaffold.[3,4] However, there has so far been very little emphasis on its characterization. This gap in understanding between structure and function is a considerable limitation for efficient scaffold design.

The difficulty with scaffolds fabricated from natural materials is that they are intrinsically variable; therefore their structural features can be difficult to characterize. Such scaffolds are frequently fabricated by a freeze-drying technique, in which the pore structure is defined by the solidification of ice crystals from an aqueous slurry of polymer fibers. Whereas a thorough understanding of the relationship between freeze-drying conditions and resulting pore size has been developed,[5] no such understanding exists for interconnectivity. Although the pore space will be predominantly interconnected, due to the characteristic interlocking of the ice crystals,[6] the difficulty is in assessing the extent of this interconnectivity, especially with relevance to cell invasion. To some extent, interconnectivity can be estimated by a visual assessment from microscopy, and in these cases, greater interconnectivity has been linked to improved cell distribution.[7,8] For more rigorous structural characterization, 3D tomographic representations of the scaffolds of interest are often used, such as those from X-ray microcomputed tomography (Micro-CT). One approach is to measure the fraction of pore space accessible from the scaffold exterior.[9,10] However, problems exist in scale-up of these measured values from Micro-CT to results that are meaningful at the scale of a bulk sample.[11] Percolation theory, which deals with the mathematical treatment of transport properties in porous solids, is a recognized solution to the problem of Micro-CT scalability.[12] It has not yet, however, been implemented for the study of cell accessibility in tissue engineering scaffolds. Whereas existing Micro-CT characterization methods focus on thorough parameterization of individual pores and fenestrations,[13,14] with this approach there is little emphasis placed on the spatial distribution of the fenestrations, and whether or not they provide continuous pathways suitable for cell invasion. The ideal method for interconnectivity assessment should include scale-independent characterization of the transport pathways through the structure, and the tools necessary for this approach may be found in percolation theory.

In this communication, we demonstrate the use of percolation theory to investigate scaffold interconnectivity in terms of a characteristic feature size for cell transport. We introduce a scale-invariant parameter, termed the “percolation diameter,” to describe the characteristics of the transport pathways encountered by an invading object. In combination with measurement of pore size, we demonstrate the relevance of the percolation diameter for predicting the extent of cell invasion. So named after the work of Saxton,[15] the percolation diameter is the size of the largest spherical object able to travel through an infinitely large scaffold. This may be considered a critical value in terms of interconnecting pathways: scaffolds with a certain percolation diameter will impede the transport of any object larger than this diameter. The methodology for its calculation, illustrated in **Figure** **1**, is based on successive measurements of *L* and *d*, where *d* is the diameter of the largest sphere able to travel a linear distance *L* through the pore space. Using a scaling relationship from percolation theory, these measurements may then be extrapolated to find the value of *d* as *L* approaches infinity: this value *d*_c_ is termed the percolation diameter.

**Figure 1:**
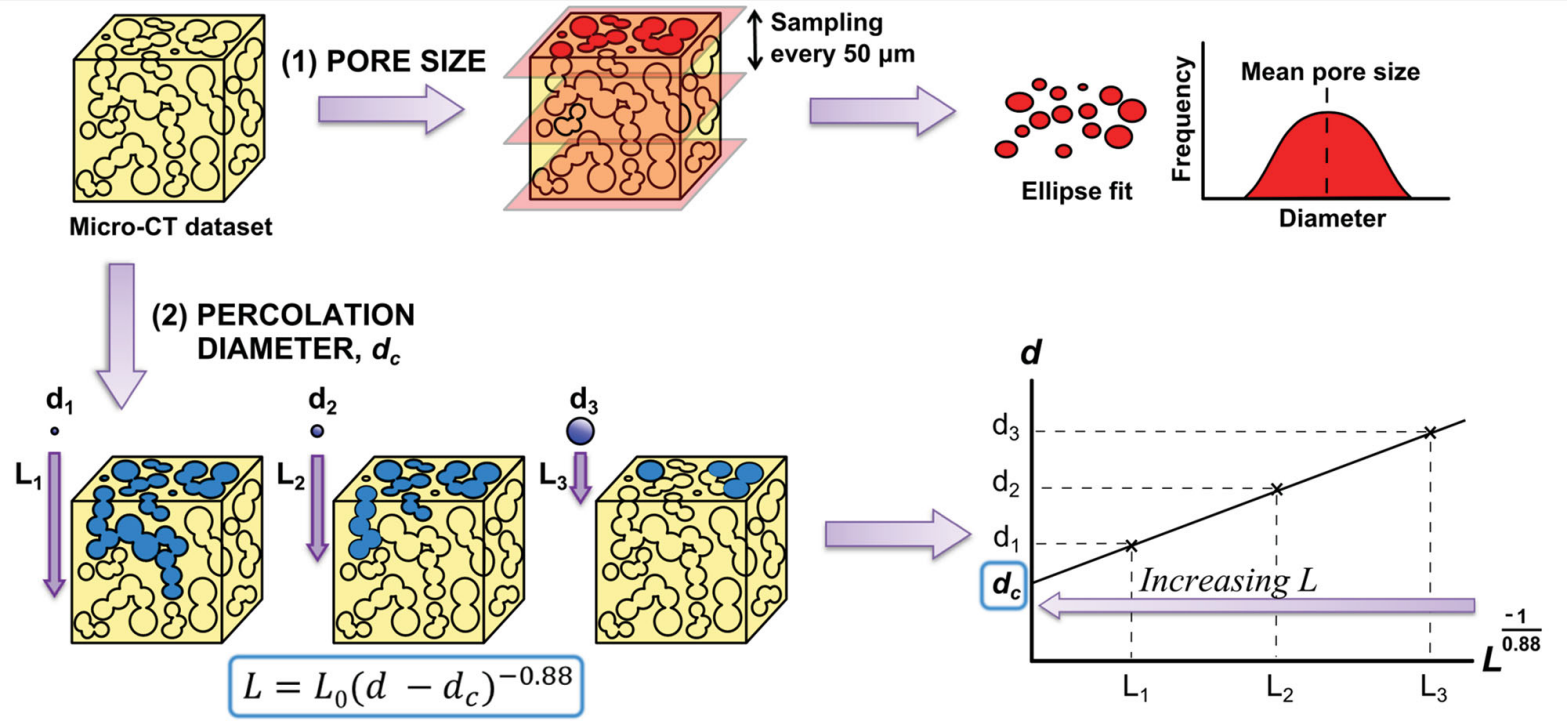
Pictorial representation of the methodology used for calculation of pore size and percolation diameter. Pore size was calculated by ellipse fit to z-slices sampled from the Micro-CT dataset. Percolation diameter was calculated by measuring the maximum accessible *z*-distance, *L*, to invading objects of varying diameter, *d*, and extrapolating to infinite scaffold sizes. Full details are given in the Experimental Section.

To demonstrate the power of this approach, a series of freeze-dried collagen scaffolds with measurable differences in structure was required, for correlation to observed biological response. Several of the variables in the freeze-drying process were investigated, to assess their potential for producing differences in scaffold interconnectivity. One of the most promising was the choice of suspension medium; a variable that has previously been shown to produce dramatic differences in scaffold architecture.[16] We chose to compare the structures obtained from two common variants of suspension medium: 0.05 m acetic acid and 0.001 m hydrochloric acid (HCl). Acetic acid is a good solvent for collagen and therefore interacts strongly with the suspended fibrils.[17] This leads to the formation of discrete pore walls, by collagen rejection at the ice crystallization interface. Conversely, the use of HCl at low concentrations is known to change the morphology of the solidifying ice.[18] We hypothesized that these effects may influence the interconnectivity of the resulting scaffolds.

Initial microstructural examination under scanning electron microscopy (SEM) revealed clear differences in pore structure according to the choice of acidic suspension medium. The SEM images in **Figure** **2** reveal the contrast between the smooth, planar pore walls obtained with acetic acid, and the much more fibrous structure obtained with HCl. This effect can be attributed to the limited interaction between HCl and the collagen fibers, resulting in fiber trapping within the solidifying ice.[18] Micro-CT analysis revealed that on the change from acetic acid to HCl, the percolation diameter decreased from 72 ± 5 to 32 ± 2 μm. The range of scaffold architectures could be extended even further by the control of additional variables, such as the suspension cooling rate: a recognized factor in determining both pore size and morphology.[19,20] By simultaneously adjusting such variables, it was found to be possible to produce a range of scaffolds with percolation diameters between 32 and 100 μm. The pore size range of these scaffolds, 52–70 μm, was comparatively small. Importantly, statistical analysis revealed that significant differences in percolation diameter were achievable in scaffolds with no significant difference in pore size (see the Supporting Information for full data). The initial part of this study therefore showed, for the first time, that pore size and interconnectivity can be independently controlled in freeze-dried collagen scaffolds.

**Figure 2:**
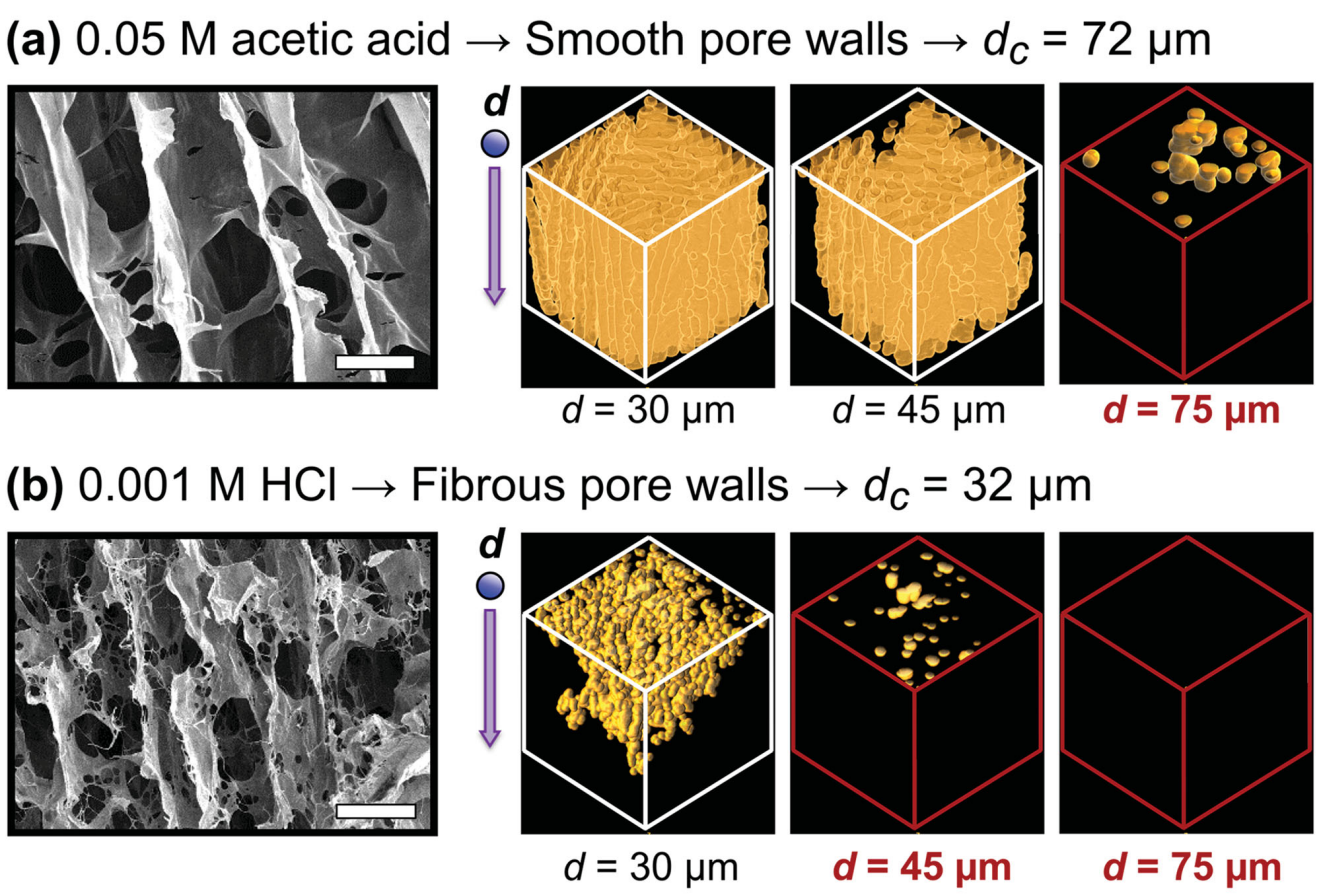
Scanning electron micrographs (left, scale bar 50 μm) and Micro-CT visualization of accessible pore space to an invading object of diameter *d* (right), for scaffolds fabricated with a) 0.05 m acetic acid and b) 0.001 m HCl. The emboldened/red-highlighted Micro-CT volumes (1 mm^3^) represent the case where the invading object is larger than the percolation diameter, *d*_c_.

The aim of the second part of the study was to assess the invasion potential of connective tissue cells in response to changes in percolation diameter. By controlling the freeze-drying conditions as discussed above, five key scaffolds were produced, with constant pore size but varying percolation diameter. Primary human fibroblasts were chosen for the cell invasion tests, as these were considered the most relevant for the intended end application of connective tissue regeneration. Three days after fibroblast seeding onto the scaffold surfaces, the scaffolds were harvested and fluorescently stained to reveal the actin cytoskeleton of the fibroblasts. Cross-sections were then taken in order to test the hypothesis that percolation diameter would have a measurable influence on cell invasion.

Representative cross-sections of each scaffold after three days of culture may be seen in **Figure** **3**. It can be seen from these images that the extent of cell invasion varies widely between the different architectures. Most notably, there appeared to be limited invasion in the two scaffolds of lowest percolation diameter. To confirm this result, we measured the intensity of actin fluorescence, *I*, as a function of distance from the scaffold surface, *Z*, as illustrated in Figure 3b for the two scaffolds previously displayed in Figure 2. A shallower decline in fluorescent intensity corresponds to a more even distribution of actin across the scaffold cross-section, and therefore more efficient cell invasion. Measured intensity values were normalized to total measured intensity, 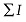, such that the integral of each intensity profile was kept constant. It can be seen from Figure 3b that percolation diameter affected not only the maximum invasion distance achieved by the cells but also the proportion of cells that remained close to the scaffold surface. To compare this invasion efficiency quantitatively across all tested scaffolds, we used these intensity plots to measure the median cell position for each scaffold. This was plotted as a function of percolation diameter, as shown in Figure 3c. This plot highlights an interesting relationship between percolation diameter and cell invasion efficiency. For scaffolds with percolation diameter greater than 40 μm, cell invasion efficiency remains roughly constant, with only a gradual possible increase in median cell position with percolation diameter. However, median cell position shows a sharp decrease for the scaffolds with percolation diameter below 40 μm. It seems, therefore, that there may be a critical interconnectivity threshold for persistent, directed cell invasion.

**Figure 3:**
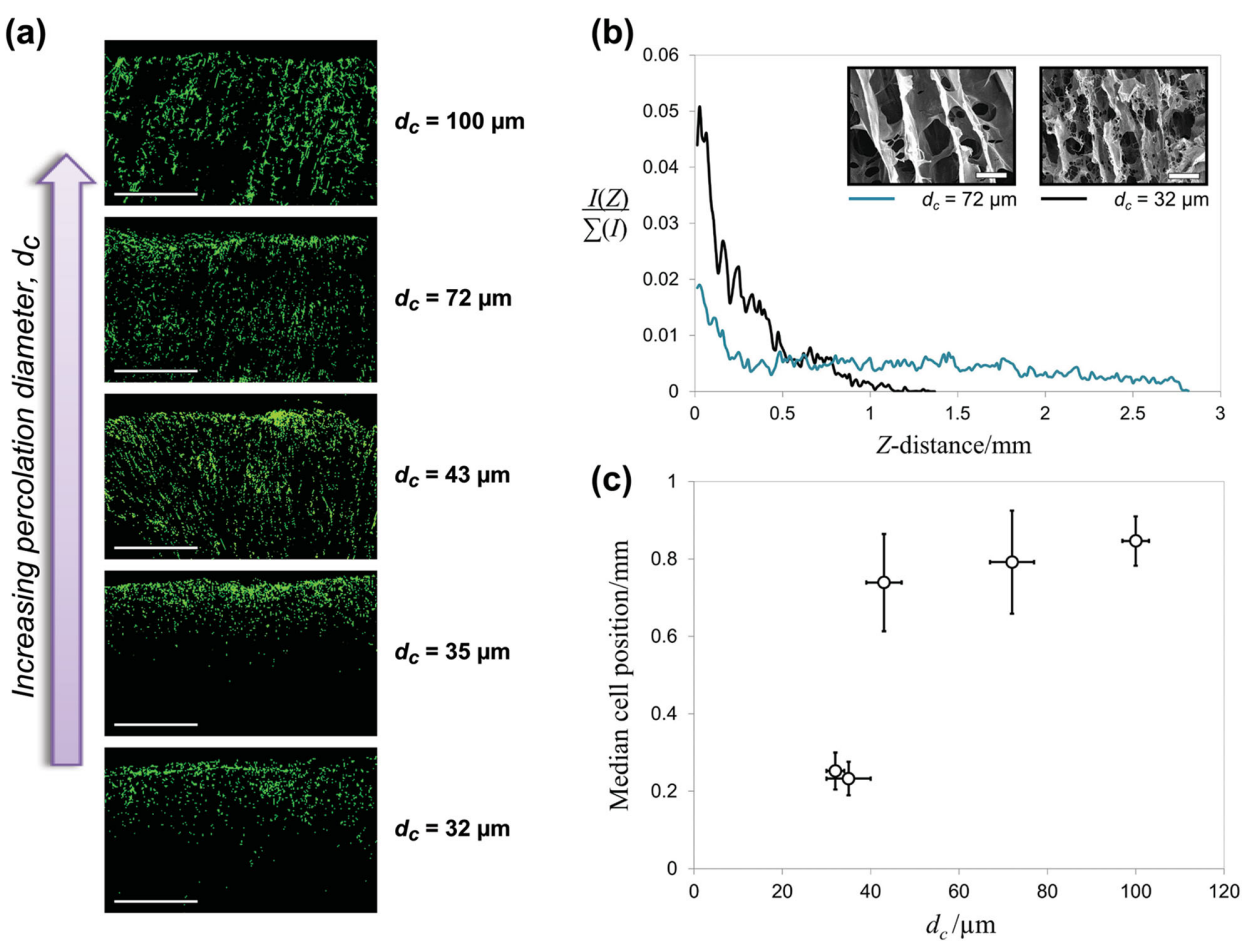
Cell invasion results after three days culture, shown a) in cross-section for scaffolds of successively increasing percolation diameters (scale bar 1 mm), b) as fluorescent intensity profiles (for the scaffolds shown in Figure 2, SEM scale bar 50 μm as before), and c) as a plot of median cell position against percolation diameter.

By isolating the effect of interconnectivity from that of pore size, we have therefore demonstrated that characterization of only one of these parameters is not sufficient for reliable prediction of cell invasion. This result has profound implications for the design of scaffolds for soft tissue engineering. It implies that optimization of pore size is an incomplete approach to the enhancement and control of cell invasion, and that an understanding of interconnectivity is required to ensure efficient cell movement into a structure. In this study, percolation diameters above 40 μm were required to ensure scaffold accessibility to connective tissue cells. It is interesting to note that in microfluidic channels, both mouse fibroblasts and human mesenchymal stromal cells show a step change in migration rate at channel widths of 40 μm.[21] Similarly, obstruction of tumor-derived (HT1080) cell migration through two-photon polymerized structures has been observed when the polymer walls making up the structure are more closely spaced than 50 μm.[22] In each case, a change in cell morphology was observed as the size of the obstructions approached the approximate length scale of a cell (10–30 μm). Importantly, whereas these studies used synthetic polymers to allow microfabrication of scaffolds with discrete feature sizes, we have implemented the percolation diameter methodology to demonstrate that similar structure–function correlations can be drawn in natural polymer scaffolds.

Percolation diameter is readily assessed using Micro-CT analysis, and thanks to its scale-invariance from percolation theory, it is relevant for the study of cell invasion in bulk samples. This novel approach will enable future investigation of the relative importance of pore size and interconnectivity, as well as the cell-type dependence of the observed critical interconnectivity threshold for cell invasion. Analysis of the largest object that may traverse the pore space has also been previously investigated using 3D confocal microscopy of collagen networks, although not in a scale-independent manner.[23] This does, however, signify that such a methodology may be implemented using datasets from a range of imaging techniques. It should be noted that for this study, percolation diameter was measured from scaffolds in the dry state. Further investigation into the effect of scaffold hydration will be an interesting extension, crucial for understanding the response of cells in culture. In addition, complementary techniques such as permeability measurement are currently being investigated in our labs, to enable comparison of fluid flow characteristics with cell invasion potential. We suggest that the percolation diameter approach could provide the means to quantify the necessary interconnectivity threshold required for soft tissue regeneration. Although interconnect sizes above 100 μm are recognized as necessary for bone tissue regeneration,[24] this is not necessarily a transferable principle to the very different application of soft tissue engineering, which often requires pore structures on a smaller scale.[25]

In summary, we have introduced a novel method for the interconnectivity characterization of collagen scaffolds, and using this method we have demonstrated the independence of interconnectivity and pore size. Using percolation diameter as a parameter for the description of scaffold interconnectivity, we have demonstrated its relevance in determining the extent of fibroblast invasion. This methodology has the potential to enable quantification of the necessary scaffold characteristics for cell invasion and soft tissue regeneration, and to help develop a more thorough understanding of the relationship between scaffold architecture and biological function. By optimization of pore size and percolation diameter, in combination with other properties such as contact guidance by collagen alignment, it is hoped that this approach will open up new strategies for inducing controllable, directed cell invasion.

## Experimental Section

*Scaffold Fabrication*: Insoluble fibrillar type I collagen from bovine Achilles tendon (Sigma-Aldrich, UK) was hydrated overnight at 1% (w/v) in either 0.05 m acetic acid (Alfa-Aesar, UK) or 0.001 m hydrochloric acid (Sigma-Aldrich). After homogenization and centrifugation for air bubble removal, the resulting collagen suspension was poured into stainless steel molds, filling height < 1 cm. The molds were placed into a VirTis AdVantage benchtop freeze-drier (Biopharma Process Systems, UK), which was either precooled to −35 °C or ramped to −35 °C from room temperature at 1.2 °C min^−1^. An additional scaffold was created in a silicone mold, filling height 2 cm, by quenching the acetic acid suspension to −20 °C. After complete freezing, a pressure of 80 mTorr and a temperature of 0 °C were maintained for ice sublimation. The resulting scaffolds were chemically cross-linked using 1-ethyl-3-(3-dimethylaminopropyl) carbodiimide hydrochloride (EDC, Sigma-Aldrich) and *N*-hydroxysuccinimide (NHS, Sigma-Aldrich), with 95% ethanol as solvent. EDC and NHS were used in the molar ratio 5:2:1 relative to the collagen carboxylic acid groups (EDC:NHS:COOH). Scaffolds were immersed in the cross-linking solution for two hours, before thorough washing with distilled water (5 × 5 min), and drying using the same freeze-drying cycle as before.

*Scaffold Image Acquisition and Pore Size Measurement*: For qualitative SEM analysis, scaffolds were sectioned in the plane containing the freezing direction (the *x*–*z* plane) using a scalpel, and sputter-coated with gold/platinum. A JEOL JSM-820 SEM was used for image acquisition, in secondary electron mode at 10 kV. For quantitative Micro-CT analysis a Skyscan 1072 system (Bruker, BE) was used to image scaffold samples cut with a 5 mm biopsy punch. Projection images were taken at 25 kV and 138 μA, with 0.23° rotation steps and 7.5 s image acquisition time, averaged over four frames. Magnification was set at 75x, pixel size 3.74 μm. Projections were processed into 3D datasets using the Skyscan reconstruction software NRecon, before binarization using the Trainable Segmentation plugin within the ImageJ software distribution FIJI. Image noise was reduced using individual z-slice despeckle, followed by a 2 × 2 × 2 median filter in 3D. Z-slices were sampled from the dataset at 50 μm spacing and mean pore size over 20 slices was calculated using FIJI: after removal of outliers larger than 2 pixels, a watershed algorithm was applied to the dataset, to allow ellipse fit to each pore. Pore size refers to the mean diameter of the circle of equivalent area to these best-fit ellipses.

*Percolation Calculations*: The median-filtered scaffold dataset was imported into the Skyscan analysis software CTAn. A cubic region of interest (ROI) was defined such that only one face of the cube, an *x*–*y* face, was accessible to invasion. Face dimensions were set at 1 mm × 1 mm (for visualization as in Figure 2) or at 2 mm × 2 mm (for numerical analysis). The CTAn function “ROI Shrinkwrap” was then used to identify the volume accessible to a virtual object. The diameter of this object, *d*, was controlled, and the corresponding length of the accessible pore volume in the *z*-direction, *L*, was measured. These measurements were then plotted according to the following relationship from percolation theory:[15]


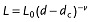


where *v* is a percolation constant with value 0.88 for 3D systems.[26] Values of *d* were plotted as a function of 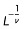 to allow calculation of the intercept: the percolation diameter, *d*_c_.

*Cell Culture*: Human periodontal ligament fibroblasts (Lonza, CH) were cultured in high glucose Dulbecco's Modified Eagle Medium (LifeTechnologies, CH) with 5% fetal bovine serum and 1% penicillin/streptomycin. Trypsin-EDTA was used to detach the subconfluent fibroblasts, which were seeded at passage five. Scaffold samples approximately 10 mm × 10 mm × 2 mm were sterilized in 70% ethanol, before washing twice in phosphate buffered saline (PBS, LifeTechnologies) and subsequent prewetting in medium. Excess medium was aspirated from the scaffolds before seeding in triplicate onto the 10 mm × 10 mm face, at a concentration of 64 000 cells in 50 μL medium per scaffold. Extra medium was added after one hour at room temperature. Culture conditions were maintained at 37 °C and 5% CO_2_ for three days, with one medium change. At day three, medium was removed and the scaffolds were washed in PBS, before fixing with 10% formalin (Sigma-Aldrich).

*Staining and Microscopy*: Once washed in PBS, scaffolds were immersed in 0.1% Triton X-100/PBS (Sigma-Aldrich) for 10 min and then washed in PBS before cytoskeletal actin staining with Alexa Fluor 488 Phalloidin (MolecularProbes, CH) at 2.5 μL/200 μL in 1% bovine serum albumin/PBS (BSA, Sigma-Aldrich). Scaffolds were then embedded in 15% gelatin/PBS (BioGel, CH), and the solidified gelatin blocks were fixed with 10% formalin. A Leica VT1000 S Vibratome was used to section these blocks at a thickness of 200 μm, to reveal the scaffold cross-section. A Yokogawa CV1000 Cell Voyager confocal microscope was used to record the maximum fluorescent intensity over 11 *z*-slices, spacing 20 μm, for each scaffold cross-section. For each scaffold condition, two biological replicates were chosen for analysis, deliberately selected such that local collagen wall orientation was kept constant between scaffold conditions. Three sections were taken from each of these replicates, giving a total of six images for study per scaffold condition. Fluorescent intensity profiles were averaged over a width of 4 mm (300 pixels) and background intensity values (measured from an empty area of the image) were subtracted. Measured intensity values *I* were normalized to the total summed intensity over the profile, ∑*I*, and the median cell position was calculated by finding the distance at which the cumulative intensity equaled half the total summed intensity.

*Statistics*: For pore size and percolation diameter, the mean of three measurements was calculated, along with standard error of the mean. For median cell position, the mean and standard error of six measurements was calculated. Statistical significance was tested using one-way ANOVA and Games-Howell analysis was used for pairwise comparisons (significance level *p* < 0.05).

## Supporting Information

As a service to our authors and readers, this journal provides supporting information supplied by the authors. Such materials are peer reviewed and may be re-organized for online delivery, but are not copy-edited or typeset. Technical support issues arising from supporting information (other than missing files) should be addressed to the authors.
