# Supplementary material for: Cell Invasion in Collagen Scaffold Architectures Characterized by Percolation Theory
Source: Adv Healthc Mater. 2015 Apr 16;4(9):1317–21. doi: 10.1002/adhm.201500197 (PMC4529738; doi:10.1002/adhm.201500197)
Supplement: Supplementary file 1 — Supplementary [file adhm0004-1317-sd1.pdf]

Copyright WILEY-VCH Verlag GmbH & Co. KGaA, 69469 Weinheim, Germany, 2015.

ADVANCED HEALTHCARE MATERIALS

Supporting Information

for *Adv. Healthcare Mater.*, DOI: 10.1002/adhm.201500197

Cell Invasion in Collagen Scaffold Architectures Characterized by Percolation Theory

*Jennifer C. Ashworth, Marco Mehr, Paul G. Buxton, Serena M. Best, and Ruth E. Cameron**

Copyright WILEY-VCH Verlag GmbH & Co. KGaA, 69469 Weinheim, Germany, 2013.

Supporting Information

Cell Invasion in Collagen Scaffold Architectures Characterized by Percolation Theory

*Jennifer C. Ashworth, Marco Mehr, Paul G. Buxton, Serena M. Best and Ruth E. Cameron**

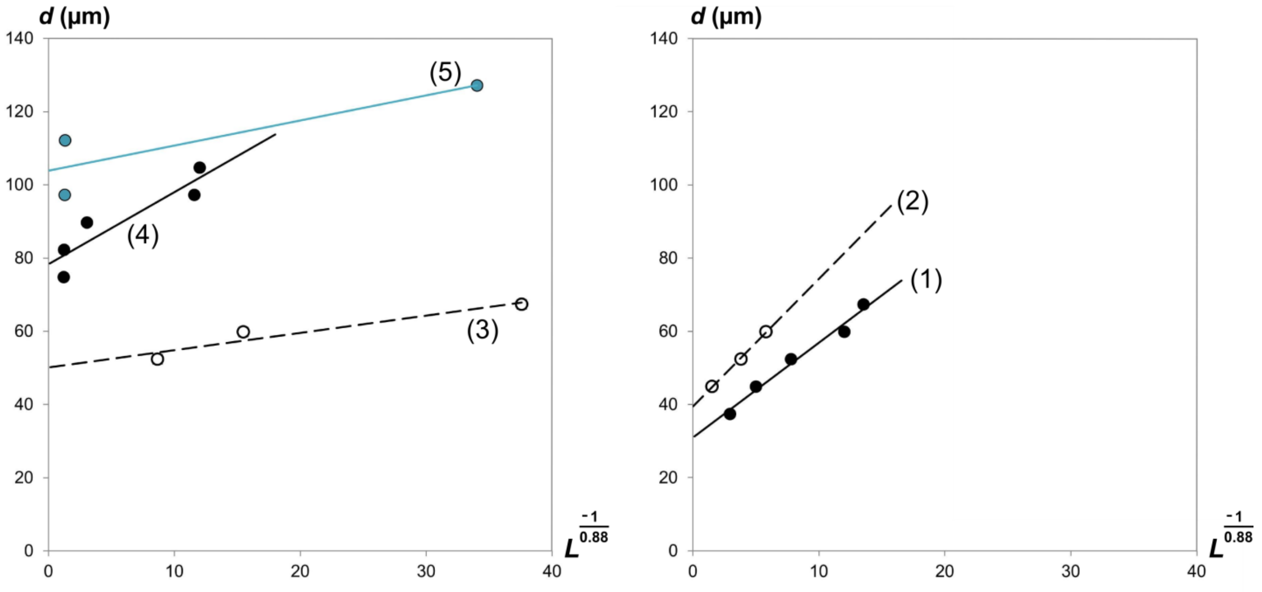

S1. Example measurements of L and d for each scaffold condition, plotted according to the percolation relationship $L = L_0(d - d_c)^{-0.88}$. The intercept of each plot corresponds to one measurement of the percolation diameter, d_c. Data for scaffolds fabricated using (a) 0.05 M acetic acid and (b) 0.001 M HCl as suspension medium are plotted separately for clarity, but on the same scale for ease of comparison. Individual plots are labelled to enable cross-reference with the scaffold parameters displayed in S2 and S3.

S2. Table of percolation diameters for the five key scaffolds tested for cell invasion efficiency, along with a brief description of the key fabrication conditions. 1% (w/v) collagen suspension with suspension medium as indicated was frozen in a steel mold (*silicone mold). Mean is given ± standard error of three measurements from different regions of each sample.

	**Percolation diameter (μm)**	**Suspension medium**	**Freezing condition**
(1)	32 ± 2	0.001 M HCl	1.2°C min^{-1} to -35°C
(2)	35 ± 5	0.001 M HCl	Quench to -35°C
(3)	43 ± 4	0.05 M acetic acid	Quench to -35°C
(4)	72 ± 5	0.05 M acetic acid	1.2°C min^{-1} to -35°C
(5)	100 ± 3	0.05 M acetic acid	Quench to -20°C *

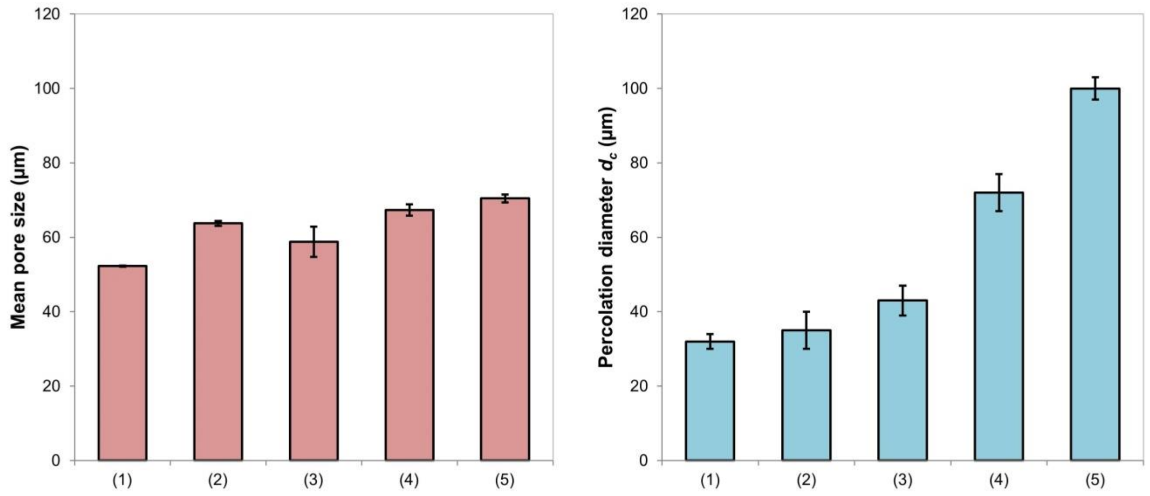

S3. Bar charts displaying the pore size and percolation diameter ranges, calculated using the methodology from figure 1, of the five key scaffolds tested for cell invasion efficiency. The pore size differences between scaffolds (2), (3) and (4) were not statistically significant ($p > 0.05$). However, due to the intrinsic variability of freeze-dried scaffolds it was not always possible to produce scaffolds with identical pore size. Therefore scaffold (1) contained significantly smaller pores than all scaffolds except (3). Although the pore size of scaffold (5) was significantly larger than that of scaffolds (1) and (2), note the comparatively small variation compared with the changes in percolation diameter. For both parameters, mean is shown ± standard error of three measurements.
